# The Antioxidant Enzyme PON1: A Potential Prognostic Predictor of Acute Ischemic Stroke

**DOI:** 10.1155/2021/6677111

**Published:** 2021-02-05

**Authors:** Yuzhen Xu, Kai Wang, Qian Wang, Yihong Ma, Xueyuan Liu

**Affiliations:** ^1^Department of Neurology, Shanghai Tenth People's Hospital, Tongji University School of Medicine, Shanghai, China; ^2^Department of Neurology, Second Affiliated Hospital of Xuzhou Medical University, Xuzhou, Jiangsu Province, China; ^3^Department of Central Laboratory, Taian City Central Hospital, Shandong First Medical University & Shandong Academy of Medical Sciences, Taian, Shandong Province, China; ^4^Department of Neurology, Graduate School of Medical Sciences, Kumamoto University, Kumamoto, Japan

## Abstract

**Objective:**

Paraoxonase 1 (PON1) is an antioxidant enzyme, which has been proved to be involved in the pathophysiological process of oxidative stress and various neurological diseases in recent years. Although reduced PON1 activity has been reported in patients with acute ischemic stroke (AIS), the prognostic value of PON1 in AIS has not been clearly established. The purpose of this study was to determine whether the baseline serum PON1 activity level is related to the functional outcome of AIS patients.

**Methods:**

From July 2017 to June 2020, AIS patients within 3 days of symptom onset were continuously prospectively included in the study. On admission, clinical and laboratory data were recorded, and serum PON1 activity was tested. The National Institute of Health Stroke Scale (NIHSS) score was used to evaluate the initial neurologic deficit at admission, and the modified Rankin scale (mRS) was used to evaluate the functional outcome at 3 months. A multiple logistic regression model was used to analyze the relationship between the baseline PON1 activity level and the prognosis of AIS.

**Results:**

A total of 336 AIS patients were finally included in this study. The serum PON1 activity of AIS patients with good outcomes was significantly higher than that of patients with poor outcomes (193.4 ± 16.3 U/mL vs. 127.2 ± 14.9 U/mL, *p* < 0.001). However, the comparison of other clinical and laboratory data between AIS patients with good and poor outcomes was not significant (*p* > 0.05). There was a significant decrease in the mRS score in patients with AIS across serum PON1 quartiles (3.0 ± 1.6, 2.6 ± 1.5, 2.4 ± 1.4, and 2.4 ± 1.3, *p* = 0.007). Multivariate logistic regression analysis showed that the 3-month functional outcome of AIS patients was significantly correlated with the quartile of serum PON1 activity.

**Conclusions:**

This study suggests that the serum PON1 activity may be an independent predictor of the functional outcome of AIS patients.

## 1. Introduction

Stroke is defined as any clinical syndrome of the permanent brain, spinal cord, or retinal cell death caused by vascular etiology based on pathology, imaging, or clinical evidence [1]. According to the World Health Organization (WHO), stroke is the second leading cause of death and the third leading cause of disability in the world [2]. In China, as the population ages, stroke has become the leading cause of death, accounting for about one-third of the global stroke mortality [3]. The GBD 2013 Study found that there were 10.3 million new strokes, 6.5 million people died of stroke, 113 million disability-adjusted life-years (DALYs), and 25.7 million stroke survivors in 2013 [4]. Due to the high mortality and disability rate, the economic burden of stroke is heavy [5]. In the United States, the total cost of stroke in 2008 was US$65.5 billion, and it is predicted to reach US$184.1 billion by 2030 [6]. Currently, there are few effective treatments to improve the prognosis of ischemic stroke [7, 8]. Therefore, looking for predictors of stroke is the focus of addressing public health concerns.

Paraoxonase (PON) is a type of arylesterase with ester hydrolysis properties [9]. The PON family mainly includes 3 members: PON1, PON2, and PON3 [10]. PON1 and PON3 are mainly synthesized by the liver and can be distributed in plasma, while PON2, as an intracellular enzyme, is expressed in almost all tissues [11]. Among them, PON1 is the most widely studied member of the PON family. Thus, we only considered the detection of PON1 in our current study. PON1 protein is composed of 354 amino acids and has a molecular weight of 43 kDa, which is considered to be a marker of antioxidative stress [12]. Recent studies have shown that PON1 has low substrate selectivity and can participate in the physiological processes of various diseases and is closely related to antioxidant defense and cerebrovascular health [13].

PON1, a granzyme carried by high-density lipoprotein, provides antioxidant and anti-inflammatory capabilities [14]. In recent epidemiological studies, its role as a protective factor against atherosclerosis has become more and more obvious [15]. Interestingly, in patients with dementia such as Alzheimer's disease (AD), the activity of PON1 decreases, suggesting that PON1 may play a neuroprotective effect [16]. However, there are few reports on the correlation between PON1 and the prognosis of acute ischemic stroke (AIS) [17]. The purpose of this study is to determine whether serum PON1 can be used as a predictor of AIS prognosis.

## 2. Methods

### 2.1. Study Population

**Figure 1:**
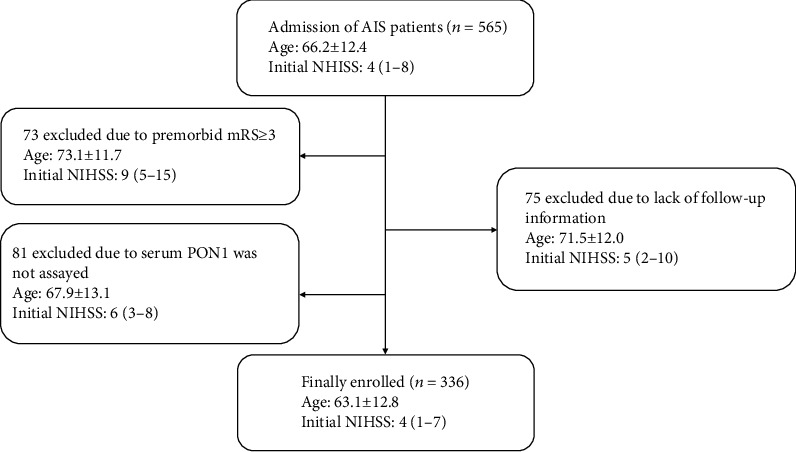
Flow chart of the study implementation. AIS: acute ischemic stroke; NIHSS: National Institute of Health Stroke Scale; PON1: paraoxonase 1.

From July 2017 to June 2020, 565 consecutive AIS patients who were admitted to the Second Affiliated Hospital of Xuzhou Medical University within 3 days of onset were screened for inclusion in our study. The definition of AIS meets the standards of the World Health Organization, and its clinical diagnosis is based on brain CT or MRI. The inclusion criteria for AIS are as follows: modified Ranking Scale (mRS) ≤ 2, within 3 days of onset, and ischemic stroke confirmed by brain CT or MRI [18]. The exclusion criteria of AIS are as follows: cerebral hemorrhage, tumor, intravenous thrombolytic therapy, arterial thrombectomy therapy, cerebrovascular interventional therapy, transient ischemic attack (TIA), and severe heart, liver, kidney, and other organic diseases [19]. Among 565 patients with AIS, 73 cases were excluded because of the modified Rankin Scale (mRS) score ≥ 3 points before the screening, and 75 cases were excluded because of lack of follow-up information. In addition, another 81 patients with AIS were excluded because they did not complete the serum PON1 activity test. Therefore, a total of 336 participants were included in this study, as shown in Figure 1. The protocol was designed according to the Declaration of Helsinki and approved by the local ethics committee. All subjects signed a written informed consent form when they were included in the study.

### 2.2. The Clinical and Laboratory Data

Upon admission, the patient's clinical data is collected through interviews with the patient or his family. And standardized experimental methods are used to detect the laboratory data. The clinical and laboratory data include age, gender, body mass index (BMI), hypertension (HP), hyperlipidemia (HLP), diabetes mellitus (DM), coronary heart disease (CHD), atrial fibrillation (AF), leukocyte, hemoglobin, total cholesterol (TC), triglycerides (TG), high-density lipoprotein cholesterol (HDL-C), low-density lipoprotein cholesterol (LDL-C), fasting blood glucose (FBG), and hemoglobin A1c (HbA1c). All the clinical and laboratory data were carefully recorded for statistical analysis.

### 2.3. Clinical Assessment

The NIH Stroke Scale (NIHSS) is used to assess the severity of stroke on admission. The mRS score was used to evaluate the functional prognosis at 3 months. At 3 months, mRS ≥ 3 points are considered poor outcomes, and mRS < 3 points are considered good outcomes. The NIHSS score is assessed by a professional neurologist, and trained researchers contact the patient or family member by telephone to assess the functional outcome (mRS score).

### 2.4. Serum PON1 Activity

All patients fasted for at least 8 hours, and then, venous blood was collected at around 7 am. The venous blood was allowed to stand at room temperature for 20-30 minutes and then centrifuged to collect the serum. The serum was aliquoted and stored in a refrigerator at -80°C for later use. Commercial reagents are used to detect the activity of PON1 on oxygenase in serum. The mechanism is to measure the activity of PON1 by measuring the change of spectrophotometry during the reaction of the enzyme substrate. The specific operation process refers to previous studies and reagent instructions [20].

### 2.5. Statistical Analysis

The results of categorical variables are expressed as *n* or percentage, and the results of continuous variables are expressed as mean ± standard deviation (SD) or median quartile. Normally distributed variables used Student's *t* test, and asymmetrically distributed variables used Mann–Whitney *U* test. The comparison between categorical variables uses the *χ*^2^ test. According to the quartile of serum PON1 activity, subjects were divided into four groups with the same sample size. In each quartile of serum PON1 activity, the functional outcomes of AIS patients were calculated. The multivariate analysis used multiple logistic regression to determine the relationship between serum PON1 activity and 3-month functional outcomes. Statistics from IBM SPSS version 23.0 (IBM Corporation, Armonk, NY) and GraphPad Prism 8.0 (GraphPad Software, La Jolla, CA) were used for statistical analysis. A *p* value <0.05 is considered significant.

## 3. Results

### 3.1. Baseline Characteristics of AIS Patients according to 3-Month Functional Outcome

**Figure 2:**
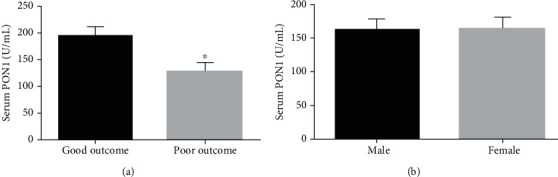
Serum PON1 activity in AIS patients. (a) The serum PON1 differences between the good outcome group and the poor outcome group (^∗^*p* < 0.05). (b) The serum PON1 differences between the male group and the female group. PON1: paraoxonase 1; AIS: acute ischemic stroke.

A total of 336 patients were included in the analysis. The clinical and laboratory data include age, gender, BMI, HP, HLP, DM, CHD, AF, leukocyte, hemoglobin, TC, TG, HDL-C, LDL-C, FBG, HbA1c, and PON1. At the end of the 3-month follow-up period, 188 patients had a good outcome (mRS 0-2), while 148 patients had a poor outcome (mRS 3-6). We also compared the clinical and laboratory data of the good outcome group and the poor outcome group. All the clinical and laboratory data are summarized in Table 1. The results showed that the clinical and laboratory data of the good outcome group and the poor outcome group, such as age, gender, BMI, HP, HLP, DM, CHD, AF, leukocyte, hemoglobin, TC, TG, HDL-C, LDL-C, FBG, and HbA1c, were not statistically different (*p* > 0.05). However, as shown in Table 1 and Figure 2(a), the serum PON1 activity of the good outcome group and poor outcome group were 193.4 ± 16.3 U/mL and 127.2 ± 14.9 U/mL, respectively. The serum PON1 activity of AIS patients in the good outcome group was significantly increased (*p* < 0.05). We further compared the effects of different genders on serum PON1 activity in AIS patients (Figure 2(b)). The results showed that the PON1 activity of male and female AIS patients was 163.9 ± 15.2 U/mL and 164.2 ± 15.7 U/mL, respectively, and there was no significant difference between them (*p* > 0.05).

### 3.2. Baseline Characteristics of AIS Patients according to PON1 Quartiles

According to the quartile of serum PON1 activity, the baseline characteristics of AIS patients are shown in Table 2. The results showed that the functional prognosis of AIS patients may have a trend relationship with the quartile of serum PON1 activity: the average mRS score of AIS patients at 3 months was lower with the increase of serum PON1 activity (*p* = 0.007). However, there is no such a trend correlation between other baseline characteristics of AIS patients and the quartile of serum PON1 activity (*p* > 0.05).

### 3.3. Association of Serum PON1 Quartiles with 3-Month Good Outcome

Logistic regression analysis of the relationship between 3-month shows good outcome and quartile of serum PON1 activity (Table 3). After controlling for age, sex, BMI, and current smoking (Model 1), compared with patients with serum PON1 Q1, patients with serum PON1 Q4 have lower mRS scores (OR: 0.531; 95% CI: 0.373-0.904; *p* = 0.014), which means that they have a better functional prognosis. After controlling for age, sex, BMI, current smoking, HP, HLP, DM, CHD, AF, leukocyte, hemoglobin, TC, TG, LDL-C, HDL-C, FBG, and HbA1c (Model 2), compared with serum PON1 Q1 patients, serum PON1 Q3 (OR: 0.639; 95% CI: 0.362-0.876; *p* = 0.024) and Q4 (OR: 0.614; 95% CI: 0.327-0.834; *p* = 0.019) patients have a better functional prognosis. After controlling age, sex, BMI, current smoking, HP, HLP, DM, CHD, AF, leukocyte, hemoglobin, TC, TG, LDL-C, HDL-C, FBG, HbA1c, and NIHSS (Model 3), compared with serum PON1 Q1 patients, serum PON1 Q3 (OR: 0.627; 95% CI: 0.391-0.814; *p* = 0.013) and Q4 (OR: 0.556; 95% CI: 0.397-0.852; *p* = 0.004) patients have a better functional prognosis. The results of the logistic regression analysis indicate that serum PON1 activity may be an independent predictor of the functional prognosis of AIS patients.

## 4. Discussion

This study explored the differences in serum PON1 activity between AIS patients with good outcome and poor outcome. The results show that AIS patients with good outcome have higher serum PON1 activity than AIS patients with poor outcome. According to the quartile levels of serum PON1, we further found that with the increase of PON1 activity, the mRS score of AIS patients after 3 months of follow-up showed a downward trend, suggesting that PON1 was related to the functional prognosis of AIS. In order to determine the correlation between PON1 and the prognosis of AIS, multiple models were introduced, and we found that PON1 may be an independent predictor of the prognosis of AIS.

PON1 is a type of granular protein arylesterase related to HDL, which is believed to have antioxidative stress properties [21]. The level of PON1 in serum mainly depends on the expression of the PON1 gene in the liver [22]. The activity of serum PON1 can be affected by genetic and environmental factors, such as atherosclerotic diet and smoking [23]. The antioxidant effect of PON1 has been confirmed in a series of diseases, such as cardiovascular disease, chronic liver disease, metabolic disease, rheumatoid arthritis, endometriosis, and childhood autism [24–29]. Interestingly, PON1 is also thought to be related to atherosclerosis and lipid peroxidation [30, 31]. However, the antiatherosclerotic mechanism of PON1 is still unclear. More and more evidence showed that the activity of PON1 was exerted by oxidizing phospholipids and homocysteine-thiolactone. Hyperhomocysteinemia and hyperthiolactoneemia caused by the imbalance of PON1 activity might be another risk factor for atherosclerosis [32]. The connection between oxidative stress and dyslipidemia established by PON1 as an intermediary suggested that it might be involved in the onset of acute ischemic stroke as a newly emerging risk factor for atherosclerosis [33]. Recently, among the Han population in China, researchers found that PON1 rs662 was a potential AIS risk, especially in males with aortic atherosclerosis [34].

PON1 exhibits a variety of physiological activities, making it an important role in the pathogenesis of neurodegenerative diseases [35]. A French study showed that serum PON1 activity was significantly decreased in vascular dementia (VaD) patients, suggesting that PON1 activity may be a biomarker for VaD patients [36]. Another Italian study showed that not only VaD but also serum PON1 activity decreased significantly in AD patients, indicating that this parameter was useful for prediction [37]. In addition, Iranian scholars have found that the onset of Parkinson's disease (PD) was not only related to the decrease of serum PON1 activity level but also related to different genotypes of PON1 [38]. The above studies suggested that PON1 might have neuroprotective effects. In recent years, studies have also confirmed that PON1 is involved in the pathogenesis of amyotrophic lateral sclerosis (ALS) and multiple sclerosis (MS) [39, 40]. The involvement of PON1 in the pathogenesis of a variety of neurodegenerative diseases suggested that more research was needed to determine its exact pathogenic mechanism and its predictive value.

In addition to neurodegenerative diseases, the role of PON1 in AIS has attracted more and more attention. The results of Ueno et al. showed that PON1 polymorphism Leu-Met55 might be involved in the development of cerebral atherosclerosis, suggesting that PON1 might be a genetic marker of cerebral infarction in Japanese [41]. A study from China found that the rs705381 and rs854571 polymorphisms in the promoter region of PON1 were closely related to the incidence of ischemic stroke in the Han Chinese [42]. The association between PON1 polymorphism and ischemic cerebral infarction has also been reported in Turkish and Spanish populations [43, 44]. The above results indicated that the polymorphism of PON1 played an important role in the pathogenesis of ischemic stroke. As a kind of lipase, in addition to gene polymorphism, the activity of PON1 is also a nonnegligible factor affecting the onset of ischemic stroke. The study of Kim et al. showed that decreased PON1 activity was a potential risk factor for AIS in Koreans [20]. Kotur-Stevuljevic and his colleagues found that the activity of PON1 in Japanese AIS patients was decreased, thereby enhancing oxidative stress, which further confirmed the role of PON1-mediated oxidative stress in AIS [45]. In China, the relationship between PON1 activity and coronary heart disease has been reported, but its relationship with AIS is still unclear [46, 47].

Our research has some limitations. First, we are a single-center small sample study. Second, our research subjects are all Chinese, so the research conclusions may not be applicable to other races. Thirdly, we did not analyze the difference of PON1 in an age-matched healthy control group and different AIS TOAST types. Fourthly, we did not detect the dynamic changes of serum PON1 activity in our current study. Fifthly, our study excluded subjects who received intravenous thrombolytic therapy, arterial thrombectomy therapy, and cerebrovascular interventional therapy, so the relationship between the prognosis of these patients and serum PON1 remains unclear. Finally, we did not detect the activity of serum PON1 in the normal population, so the trend of PON1 change under AIS is not clear. However, we are the first study in the Chinese population to study the correlation between serum PON1 activity and the prognosis of AIS, which has important clinical significance.

## 5. Conclusions

The main finding of this study is that the serum PON1 activity is related to the functional prognosis of AIS patients. Compared with AIS patients with poor outcomes, AIS patients with good outcomes have significantly higher serum PON1 activity levels and lower mRS scores. Further studies have shown that the mRS score of AIS patients tends to decrease with the increase of serum PON1 activity level. Even after adjusting for confounding factors, this correlation is still significant, suggesting that serum PON1 activity level may be an independent predictor of AIS prognosis. The relationship between serum PON1 activity level and functional prognosis of AIS patients may have important clinical and therapeutic significance if it is further confirmed in the future.

## Figures and Tables

**Table 1:** Baseline characteristics of AIS patients according to 3-month functional outcome.

Characteristics	All patients (*n* = 336)	Good outcome (*n* = 188)	Poor outcome (*n* = 148)	*p* value
Age, years	63.1 ± 12.8	62.9 ± 13.1	63.4 ± 12.4	0.722
Gender, male	196	111	85	0.766
BMI, kg/m^2^	25.0 ± 1.5	24.9 ± 1.4	25.1 ± 1.6	0.223
Current smoking	103	57	46	0.880
HP	183	101	82	0.759
HLP	126	69	57	0.733
DM	112	62	50	0.876
CHD	37	20	17	0.805
AF	56	31	25	0.922
Admission NIHSS	3 (1-7)	2 (1-3)	4 (3-7)	0.726
Leukocyte, 10^9^/L	7.5 ± 1.2	7.4 ± 1.3	7.6 ± 1.1	0.135
Hemoglobin, g/L	133 ± 16	134 ± 15	132 ± 17	0.254
TC, mmol/L	4.8 ± 1.7	4.8 ± 1.6	4.9 ± 1.8	0.591
TG, mmol/L	2.6 ± 0.7	2.6 ± 0.7	2.7 ± 0.7	0.194
HDL-C, mmol/L	1.3 ± 0.2	1.3 ± 0.2	1.3 ± 0.3	0.465
LDL-C, mmol/L	2.6 ± 0.9	2.6 ± 0.9	2.7 ± 1.0	0.336
FBG, mmol/L	7.2 ± 2.2	7.1 ± 2.0	7.3 ± 2.4	0.405
HbA1c, mmol/L	6.3 ± 1.0	6.3 ± 1.1	6.4 ± 0.9	0.371
PON1, U/mL	164.2 ± 15.7	193.4 ± 16.3	127.2 ± 14.9	<0.001

Abbreviations: AIS: acute ischemic stroke; BMI: body mass index; HP: hypertension; HLP: hyperlipidemia; DM: diabetes mellitus; CHD: coronary heart disease; AF: atrial fibrillation; NIHSS: National Institute of Health Stroke Scale; TC: total cholesterol; TG: triglycerides; HDL-C: high-density lipoprotein cholesterol; LDL-C: low-density lipoprotein cholesterol; FBG: fasting blood glucose; HbA1c: hemoglobin A1c; PON1: paraoxonase 1.

**Table 2:** Baseline characteristics of AIS patients according to PON1 quartiles.

Characteristics	Q1 (*n* = 84)	Q2 (*n* = 84)	Q3 (*n* = 84)	Q4 (*n* = 84)	*p* value
Age, years	63.3 ± 12.4	62.8 ± 13.2	63.2 ± 12.5	63.1 ± 13.1	0.995
Gender, male	46	52	47	51	0.735
BMI, kg/m^2^	25.1 ± 1.6	25.2 ± 1.6	24.8 ± 1.3	24.9 ± 1.5	0.297
Current smoking	29	24	28	22	0.608
HP	38	51	44	50	0.156
HLP	39	26	36	25	0.056
DM	23	31	25	33	0.303
CHD	10	8	12	7	0.617
AF	11	17	10	18	0.232
Leukocyte, 10^9^/L	7.6 ± 1.4	7.4 ± 1.0	7.7 ± 1.3	7.3 + 1.1	0.128
Hemoglobin, g/L	133 ± 17	134 ± 14	132 ± 18	133 ± 15	0.885
TC, mmol/L	4.8 ± 1.8	4.7 ± 1.5	4.9 ± 1.9	4.8 ± 1.6	0.902
TG, mmol/L	2.5 ± 0.7	2.7 ± 0.8	2.5 ± 0.6	2.7 ± 0.7	0.081
HDL-C, mmol/L	1.3 ± 0.2	1.3 ± 0.3	1.3 ± 0.2	1.3 ± 0.2	0.999
LDL-C, mmol/L	2.4 ± 0.7	2.7 ± 1.0	2.5 ± 0.8	2.8 ± 1.1	0.219
FBG, mmol/L	7.4 ± 2.4	7.0 ± 2.1	7.5 ± 2.3	6.9 ± 2.0	0.342
HbA1c, mmol/L	6.1 ± 0.8	6.4 ± 1.1	6.2 ± 1.0	6.5 ± 1.1	0.417
Admission NIHSS	3 (1-5)	3 (1-7)	3 (1-5)	3 (1-6)	0.236
mRS at 90 days	3.0 ± 1.6	2.6 ± 1.5	2.4 ± 1.4	2.4 ± 1.3	0.007

Abbreviations: AIS: acute ischemic stroke; BMI: body mass index; HP: hypertension; HLP: hyperlipidemia; DM: diabetes mellitus; CHD: coronary heart disease; AF: atrial fibrillation; TC: total cholesterol; TG: triglycerides; HDL-C: high-density lipoprotein cholesterol; LDL-C: low-density lipoprotein cholesterol; FBG: fasting blood glucose; HbA1c: hemoglobin A1c; NIHSS: National Institute of Health Stroke Scale; mRS: modified Rankin Scale; PON1: paraoxonase 1.

**Table 3:** Association of serum PON1 quartiles with a 3-month good outcome.

Serum PON1	OR (95% CI), *p* value			
	Q1 (*n* = 84)	Q2 (*n* = 84)	Q3 (*n* = 84)	Q4 (*n* = 84)
Model 1	1 (reference)	0.903 (0.612-0.931) *p* = 0.173	0.674 (0.350-0.926) *p* = 0.059	0.531 (0.373-0.904) *p* = 0.014
Model 2	1 (reference)	0.846 (0.358-0.910) *p* = 0.241	0.639 (0.362-0.876) *p* = 0.024	0.614 (0.327-0.834) *p* = 0.019
Model 3	1 (reference)	0.718 (0.303-0.859) *p* = 0.108	0.627 (0.391-0.814) *p* = 0.013	0.556 (0.397-0.852) *p* = 0.004

Model 1 includes age, sex, BMI, and current smoking. Model 2 further includes HP, HLP, DM, CHD, AF, leukocyte, hemoglobin, TC, TG, LDL-C, HDL-C, FBG, and HbA1c. Model 3 further includes NIHSS. PON1: paraoxonase 1; BMI: body mass index; HP: hypertension; HLP: hyperlipidemia; DM: diabetes mellitus; CHD: coronary heart disease; AF: atrial fibrillation; TC: total cholesterol; TG: triglycerides; HDL-C: high-density lipoprotein cholesterol; LDL-C: low-density lipoprotein cholesterol; FBG: fasting blood glucose; HbA1c: hemoglobin A1c; NIHSS: National Institute of Health Stroke Scale; mRS: modified Rankin Scale.

## Data Availability

The data used to support the findings of this study are available from the corresponding author upon request.
